# Impact of interventions including vaccination against *Neisseria meningitidis* on the frequency of meningitis in the African meningitis belt: a scoping review protocol

**DOI:** 10.12688/f1000research.21164.1

**Published:** 2019-11-15

**Authors:** Niurka Molina, Greissi Justiniani, Lisset Urquiza, Maria Eugenia Toledo, Chukwuemeka Onwuchekwa, Kristien Verdonck, Ermias Diro, Nivaldo Linares-Pérez

**Affiliations:** 1 Instituto de Medicina Tropical Pedro Kourí, Havana, Cuba; 2 Instituto Finlay de Vacunas, Havana, Cuba; 3 Institute of Tropical Medicine, Antwerp, Belgium; 4 University of Gondar, Gondar, Ethiopia

**Keywords:** Neisseria meningitidis, vaccination, health impact assessment, scoping review

## Abstract

In the African meningitis belt (region from Senegal to Ethiopia), there are around 30,000 reported cases of meningococcal disease per year. The main aetiological agent is *Neisseria meningitidis *of serogroup A. Since 2010, vaccination efforts have increased and hundreds of millions of people have been vaccinated. There are indications that the epidemiology of meningococcal disease is changing. This is the protocol of a scoping review, the objective of which is to describe the extent and nature of the research evidence about the impact of vaccination on meningitis frequency. Primary studies and reviews are eligible for inclusion in the review if they assess the impact of interventions that include *N. meningitidis* vaccination in countries of the African meningitis belt, report meningitis frequencies, and include an element of comparison. The sources of records are electronic databases (MEDLINE, Cochrane register of clinical trials, African Index Medicus, and clinicaltrials.gov), surveillance reports at country level, online resources of large stakeholders involved in vaccination, reference lists of included records, and experts in the field. The search strategy is based on the combination of the condition of interest, the intervention, and the geographical region. The findings of this review will be presented using figures, tables, and thematic narrative synthesis. This review will not produce a pooled estimate of what the impact of vaccination is, but will give insight in how the authors of the included records assessed the impact.

## Introduction

*Neisseria meningitidis* is a Gram-negative bacterium that is found in the mucous membrane of the nasopharynx and tonsils of about 10% of the human population ^1^. Most *N. meningitidis* strains are harmless, but some encapsulated clones are virulent and can cause meningococcaemia, meningitis, and septic shock ^1^. Historically, the highest incidence of meningococcal disease has been described in sub-Saharan Africa, in the so-called African meningitis belt, which stretches from the west of Senegal to the east of Ethiopia ^2^. In this region, endemic rates are high, and large-scale epidemics have occurred every 8–12 years for more than a century, typically in dry seasons ^1– 4^. The number of cases of meningococcal meningitis reported from the African meningitis belt is around 30,000 per year ^4^.

There are at least 13 serogroups of *N. meningitidis* (A, B, C, D, E, H, I, K, L, W135, X, Y, and Z) that are classified based on differences in capsule polysaccharides ^1^. Serogroup A used to be the main causative agent of epidemics in Africa, but massive vaccination campaigns are changing the epidemiology ^3, 5^. Validated and licensed conjugate vaccines are available for serogroups A (MenAfriVac ^®^) and C, and there is also a tetravalent vaccine for serogroups A, C, Y, and W135 ^4, 6^. These vaccines can be used in routine settings (part of routine immunisation scheme) and in response to outbreaks (reactive vaccination) ^4^. Vaccination efforts intensified in 2010 and since then, hundreds of millions of Africans have received a dose of MenAfriVac ^®^^3^. As a consequence, the incidence of meningococcal meningitis due to serogroup A has decreased, but outbreaks of new clones have been reported ^3, 5^. 

Our main objective is to evaluate the impact of vaccination on morbidity and mortality due to meningococcal disease in countries of the African meningitis belt. Before engaging in a systematic review, we will assess the size and scope of the body of literature ^6^. The aim at this stage is not to produce a pooled estimate of what the impact of vaccination is, but to evaluate how the authors of the included records have assessed the impact.

## Objectives

The central question of this scoping review is: what is the extent and the nature of the research evidence about the impact of interventions including vaccination against *Neisseria meningitidis* on the frequency of meningitis in the African meningitis belt? The review question is formulated using the SPICE (setting, perspective, intervention, comparison, evaluation) framework and the key elements are summarised in Table 1 ^7^.

**Table 1.** Overview of the key elements of the review question.

Key element	Elaboration
Setting	African meningitis belt where efforts to reduce the burden of meningitis due to *N. meningitidis* through vaccination have increased since 2010.
Perspective	Residents in the region who may benefit from vaccination.
Intervention	Interventions at individual or group level including *N. meningitidis* vaccination, combined or not with chemoprophylaxis for contacts, health information/education, other vaccines, etc.
Comparison	Subgroups with and without intervention, populations before and after intervention.
Evaluation	Impact in terms of a reduction in the frequency of meningitis or in the proportion of meningitis due to *N. meningitidis*.

## Methods

### Eligibility criteria

Records will be included in the review if they meet all the following criteria:

- Reports of primary studies or review articles (not opinion papers); and
- About people living in one of 27 countries corresponding to the African meningitis belt (any population group, any age); and
- Assessing the impact of interventions that include *N. meningitidis* vaccination; and
- Including an element of comparison (populations with *versus* without vaccination, or before *versus* after vaccination); and
- Reporting meningitis frequency. The reported condition can be meningitis due to *N. meningitidis*, meningitis in general, or death due to meningitis. Disease frequency can be expressed as absolute number of cases, prevalence, or incidence. The denominator can be the general population or a subgroup (e.g. meningitis patients).

Records reporting the impact of mixed interventions (vaccination for *N. meningitidis* + other interventions such as chemoprophylaxis to prevent meningococcal disease among contacts or vaccination for other pathogens) will be included. If we find a record that reports findings both from countries inside and outside the African meningitis belt, we will include that record and extract only that part of the data that comes from one of the 27 target countries for this review.

A sheet with detailed eligibility criteria will be used for record screening (based on titles and abstracts) and selection (based on full-text papers). A preliminary version of this sheet is available as extended data ^8^. The detailed selection criteria will be pilot-tested on 50 titles and abstracts and refined if necessary.

### Information sources

We will search the following electronic databases: MEDLINE, the Cochrane register of clinical trials, African Index Medicus, and clinicaltrials.gov. Other sources of information will be surveillance reports at country level and online resources of the World Health Organization and other large stakeholders involved in vaccination campaigns (to be identified via the included records). Finally, we also intend to screen the reference lists of included records (especially review papers) and contact experts in the field to check if we have missed any potentially relevant records. There will be no restrictions regarding language, publication date, or study design.

### Search strategy

**Figure 1.**
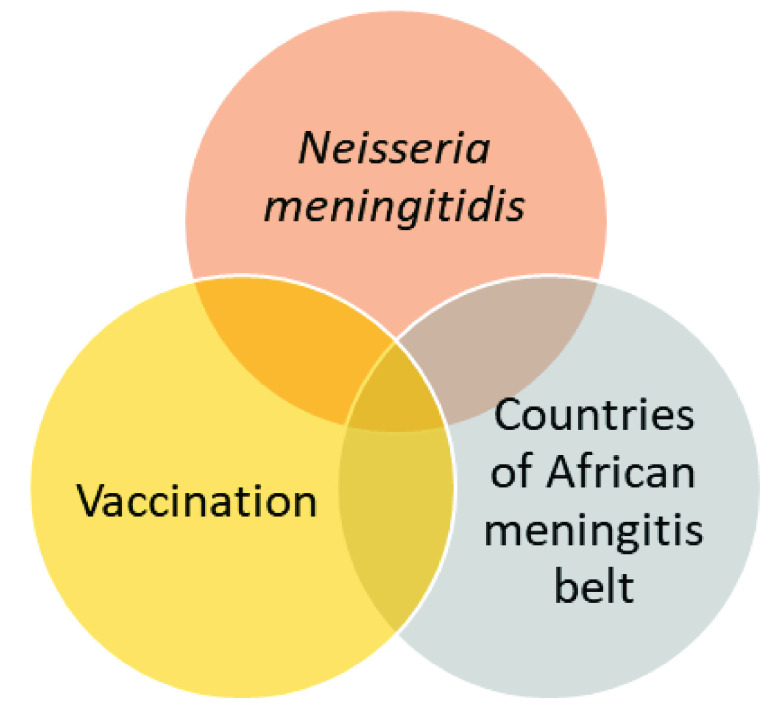
Summary of search strategy.

The search strategy is based on the combination of three concepts: the condition of interest, the intervention, and the geographical region ( Figure 1). The Boolean operators “AND” and “OR” are used to combine search terms. The planned search syntax for PubMed is given in Table 2. The same general strategy will be used to search the other databases, but small adjustments will be made such as the translation of key words to French, and the adaptation of truncation symbols and parentheses to different search engines. 

**Table 2.** Planned search syntax for PubMed.

(neisseria meningitidis[Mesh]) OR ((Neisseria) AND meningit*) OR (meningoc*)
AND
(Vaccination [Mesh]) OR (Vaccines [Mesh]) OR (Vaccinati*) OR (Vaccine*) OR (Immuni*)
AND
“Benin” [Mesh] OR “Burkina Faso” [Mesh] OR “Burundi” [Mesh] OR “Cote d'Ivoire” [Mesh] OR “Cameroon” [Mesh] OR “Central African Republic” [Mesh] OR “Chad” [Mesh] OR “Democratic Republic of the Congo” [Mesh] OR “Eritrea” [Mesh] OR “Ethiopia” [Mesh] OR “Gambia” [Mesh] OR “Ghana” [Mesh] OR “Guinea” [Mesh] OR “Guinea-Bissau” [Mesh] OR “Kenya” [Mesh] OR “Mali” [Mesh] OR “Mauritania” [Mesh] OR “Niger” [Mesh] OR “Nigeria” [Mesh] OR “Rwanda” [Mesh] OR “Senegal” [Mesh] OR “South Sudan” [Mesh] OR “Sudan” [Mesh] OR “Tanzania” [Mesh] OR “Togo” [Mesh] OR “Uganda” [Mesh] OR “Benin” [All fields] OR “Burkina Faso” [All fields] OR “Burundi” [All fields] OR “Cote d'Ivoire” [All fields] OR “Cameroon” [All fields] OR “Central African Republic” [All fields] OR “Chad” [All fields] OR “Democratic Republic of the Congo” [All fields] OR “Eritrea” [All fields] OR “Ethiopia” [All fields] OR “Gambia” [All fields] OR “Ghana” [All fields] OR “Guinea” [All fields] OR “Guinea-Bissau” [All fields] OR “Kenya” [All fields] OR “Mali” [All fields] OR “Mauritania” [All fields] OR “Niger” [All fields] OR “Nigeria” [All fields] OR “Rwanda” [All fields] OR “Senegal” [All fields] OR “South Sudan” [All fields] OR “Sudan” [All fields] OR “Tanzania” [All fields] OR “Togo” [All fields] OR “Uganda” [All fields] OR “Congo” [All fields] Or “Zaire” [All fields] OR “Guinea Bissau” [All fields] OR “Côte d'Ivoire” [All fields]

### Study records

***Data management***. Retrieved records will be automatically exported to Microsoft Excel if possible (e.g. from PubMed) and manually added otherwise. All records will get a unique identifier. Information extracted from the included records will be stored in the Excel file. Records that remain after title and abstract screening will also be kept in an EndNote file.

***Selection process***. Record screening and selection will be done in duplicate by two independent members of the review team (LU and/or GJ and/or NM). Any discordances during screening of titles and abstracts or full-text papers will be solved through discussion with a third member of the review team (KV). For each full-text record that we exclude, the main reason for exclusion will be recorded. The search and selection process will be documented in a PRISMA flowchart ^9^.

***Data collection process***. Two members of the review team (LU and/or GJ and/or NM) will independently extract the information from the included records using a standard form (preliminary version available as extended data ^8^). This data extraction form will be piloted on at least three full-text records and refined if necessary. The extracted information will first be filled out on the data extraction form (one form per reviewer and per record) and then passed to the Excel file. In case the information is unclear or incomplete, we will describe it as such; we do not intend to contact investigators. Any discordances between the two reviewers will be discussed with a third reviewer (KV).

### Data items

We will collect information about the record itself and about the study described in the record. Table 3 gives an overview of the data items. The complete preliminary data extraction form is available as extended data ^8^.

**Table 3.** Summary of data items to extract.

Characteristics of	Data items to extract
Record	Publication year; journal; publication type; last name of first author; affiliations of first, last, and corresponding author
Study	Study period; funding source; stakeholders or implementers
Setting	Context and reason for study; circulating serogroups of *N. meningitidis* as mentioned by the authors; routine *versus* research setting; study objective as formulated by the authors
Population	Country and geographical region where study took place; type and size of population undergoing intervention; type and size of population not undergoing intervention
Intervention	Type and provider of vaccine; description of intervention; intervention at individual and/or group level; objective of intervention
Impact assessment	Definition and operationalisation of impact as formulated by the authors
Study design	As formulated by the authors; as defined by the review team; elements needed for assessment of risk of bias
Evaluation	Measure of evaluation of impact as described by the authors; reported condition (meningitis due to *N. meningitidis*, meningitis in general, and/or death due to meningitis); measure of disease frequency (number of cases, prevalence, and/or incidence); denominator (general population, meningitis patients, other subgroup)

### Outcomes and prioritization

Rather than focusing on one or a few specific outcomes, this review focuses on the size and scope of the available research literature and on the nature and extent of the research evidence. The approach will be descriptive; we do not foresee outcome prioritization.

### Risk of bias in individual studies

The assessment of risk of bias will be done at study level and independently by two people of our review team. As we are using broad eligibility criteria for this scoping review, we expect to include information in heterogeneous formats and coming from studies following different designs. For randomised trials of interventions, we plan to use the risk-of-bias assessment tool of the Cochrane Collaboration, and for non-randomised studies the ROBINS-I tool. For studies following other designs, we will only describe the study design and the ways impact of vaccination was described and assessed. We plan to describe and discuss the findings of the assessment of risk of bias and will not use them in any other way in data synthesis.

### Data synthesis

The findings of this review will be presented using figures, tables, and thematic narrative synthesis. Data from the included studies will not be pooled and the synthesis will not lead to recommendations on vaccination for *N. meningitidis*.

A preliminary structure of the results section is given below, but this may slightly change depending on the content of the included papers:

- Search and selection (with PRISMA flowchart)
- Characteristics of included records: publication type, year, journal, author affiliations
- Study populations: country, setting, size, general population or subgroups
- Interventions: vaccination alone or in combination, rationale, in outbreak, routine or research settings, by government or others
- Vaccines used: type, brand, provider
- Approaches to assess impact: overview of definitions and operationalisation of impact
- Study design: according to the study authors and according to the review team
- Risk of bias assessment

## Reporting and registration

The present review protocol was developed following the Preferred Reporting Items for Systematic review and Meta-Analysis (PRISMA) guidelines, more specifically the checklist for review protocols (PRISMA-P 2015) and the extension for scoping reviews (PRISMA-ScR 2018) ^9– 11^.

The review protocol will be published so that it is publicly available before the actual reviewing activities start. PROSPERO is a specialized platform for review protocols but does not accept scoping reviews. We therefore publish the current protocol on F1000Research, an open access scientific publishing platform. Any changes in the reviewing activities after protocol registration will be listed in the final review paper.

## Planning

- Protocol publication: November 2019
- Search, selection, data extraction and synthesis: November 2019 – February 2020
- Writing of review paper: February 2020 – April 2020

## Review team and roles

The review team is presented in Table 4.

**Table 4.** Review team, affiliations, and roles.

Name	Affiliation	Role
Niurka Molina niurka.molina@ipk.sld.cu	Instituto Pedro Kourí Havana, Cuba	Write draft protocol, search & select studies, extract & synthesise data, write draft review
Greissi Justiniani gjustiniani@finlay.edu.cu	Instituto Finlay Havana, Cuba	Write draft protocol, search & select studies, extract & synthesise data, write draft review
Lisset Urquiza lurquiza@finlay.edu.cu	Instituto Finlay Havana, Cuba	Write draft protocol, search & select studies, extract & synthesise data, write draft review
Maria Eugenia Toledo mariaeugenia@ipk.sld.cu	Instituto Pedro Kourí Havana, Cuba	Provide topic expertise, interpret findings, give feedback on draft texts
Chukwuemeka Onwuchekwa emyonwuchekwa@gmail.com	Institute of Tropical Medicine, Antwerp, Belgium	Provide context knowledge, give methodological input, give feedback on draft texts
Kristien Verdonck tverdonck@itg.be	Institute of Tropical Medicine Antwerp, Belgium	Write draft protocol, give methodological input, solve discordances in study selection & data extraction, write draft review, corresponding author
Ermias Diro ermi_diro@yahoo.com	Gondar University, Gondar, Ethiopia	Provide context knowledge & clinical expertise, give methodological input, give feedback on draft texts
Nivaldo Linares-Pérez nlinares@finlay.edu.cu	Instituto Finlay Havana, Cuba	Propose topic, provide topic expertise, interpret findings, give feedback on draft texts

## Study status

While preparing the present protocol, we tried out preliminary searches to get an idea of the size of the available literature. At the time of submission, formal reviewing activities had not started yet.

## Data availability

### Underlying data

No underlying data are associated with this study.

### Extended data

Figshare: SupplementaryInformation_Eligibility_DataExtraction. https://doi.org/10.6084/m9.figshare.10078928.v1 ^8^.

Data are available under the terms of the Creative Commons Attribution 4.0 International license (CC-BY 4.0).
