# Supplementary material for: Impact of interventions including vaccination against Neisseria meningitidis on the frequency of meningitis in the African meningitis belt: a scoping review protocol
Source: F1000Res. 2019 Nov 15;8:1922. [Version 1] doi: 10.12688/f1000research.21164.1 (PMC8921686; doi:10.12688/f1000research.21164.1)
Supplement: Peer review history

# Peer review: Reviewer response for version 1

**DOI:** 10.5256/f1000research.23296.r121240

**Published:** 2022-03-14

**Authors:** Charlene M Kahler

**Affiliations:** 1 Marshall Centre for Infectious Disease Research and Training, School of Biomedical Sciences, University of Western Australia, Crawley, Western Australia, 6009, Australia

**General comments** 

 The scoping review is well written and should achieve the outcomes stated.

**Minor comments**

 Serogroup B disease is an issue in Africa and is increasing in frequency by causing local sporadic clusters, so this should be added to your search terms. Meningococcal disease is only one of many agents causing meningitis, so please be sure that your search terms can make this distinction or try to find studies that use follow up steps to confirm the causative agent. All cases of meningitis are treated the same - with ceftriaxone or penicillin, so you cannot really dissect useful information using this as a term to extract this information.

 Overall, I look forward to the final outcome of this study as a review on this topic has not been done for some time.

Is the study design appropriate for the research question?

Yes

Is the rationale for, and objectives of, the study clearly described?

Yes

Are sufficient details of the methods provided to allow replication by others?

Yes

Are the datasets clearly presented in a useable and accessible format?

Yes

Reviewer Expertise:

Meningococcal disease, meningococcal genomic epidemiology

I confirm that I have read this submission and believe that I have an appropriate level of expertise to confirm that it is of an acceptable scientific standard.

# Peer review: Reviewer response for version 1

**DOI:** 10.5256/f1000research.23296.r63667

**Published:** 2020-06-03

**Authors:** James M. Stuart, Clare French

**Affiliations:** 1 Population Heath Sciences, Bristol Medical School, University of Bristol, Bristol, UK

**General comments**

 This is a clearly written protocol for a scoping review to describe the extent and nature of the research evidence about the impact of vaccination on meningitis frequency. We note that this is being conducted before the research team embark on a full systematic review on the topic.

****

**Introduction**

 Importantly the numbers of cases (30,000 a year) quoted refer to suspected cases due to any cause, not specifically meningococcal meningitis. I note that the WHO reference to key facts is misleading on this point. Most reported cases are not laboratory confirmed. The majority of confirmed cases are meningococcal, but a substantial proportion are pneumococcal meningitis ^1^.

 The authors should emphasize this in the protocol and clarify how/that they will take account of information on laboratory confirmation of meningococcal and other pathogens when interpreting the data obtained. This is clearly important in assessing the impact of meningococcal vaccines and the validity of the review.

**Minor:**

 There are currently 12 (not 13) serogroups, and W135 is no longer used, it has been replaced by W ^2^.

 In the abstract, not in the main text, it is stated that Serogroup A is the main aetiological agent of meningitis in the belt. This was true until 2010, but not since.

 There are 26 (not 27) countries in the meningitis belt according to WHO https://www.who.int/gho/epidemic_diseases/meningitis/en/. 

1. Trotter, C.L., et al., *Impact of MenAfriVac in nine countries of the African meningitis belt, 2010-15: an analysis of surveillance data.* Lancet Infect Dis, 2017. **17**(8): p. 867-872. ^1^.
2. Harrison, O.B., et al., *Description and nomenclature of Neisseria meningitidis capsule locus.* Emerg Infect Dis, 2013.# **19**(4): p. 566-73. ^2^. 

**Methods**

 The authors plan to search various sources in addition to electronic databases but they don’t mention searching conference abstracts. Though this is not essential for a scoping review it could be another way of identifying work in progress – such work may well have been published by the time the authors conduct their planned systematic review on the topic so it might be helpful to include it at this stage.

 The search strategy looks fine for the purpose of a scoping review. The authors might consider adding the following Mesh term: ‘Meningococcal Infections’.

 The authors state that they will include both primary studies and review articles. Some clarification is required as to how they will avoid reporting on individual studies more than once (i.e. where an eligible individual study is identified and that study is also included in an eligible systematic review) - will reviews be used as a means of identifying individual studies to report on or will they be reported on at the review level?

 It sounds as though the search hits will be screened for eligibility in Excel. This is fine if it is the authors’ preference but an alternative could be to use free screening software such as Rayyan which can help organise and speed up the screening process ( https://rayyan.qcri.org/welcome).

 The authors state that they will assess the risk of bias for individual studies included in their review. Although this will be an important element of the full systematic review planned by the authors it is not necessarily required for a scoping review e.g. see the PRISMA extension for scoping reviews guidance on this: https://www.acpjournals.org/doi/10.7326/M18-0850. It is not clear that an assessment of risk of bias is required here. We suggest the authors give careful consideration as to whether this step is necessary, particularly given that it can be quite time consuming, and what it will add to the scoping review. If the authors do feel that risk of bias assessment is required, they should provide clear reasoning for this in relation to the review objectives.

Is the study design appropriate for the research question?

Yes

Is the rationale for, and objectives of, the study clearly described?

Yes

Are sufficient details of the methods provided to allow replication by others?

Yes

Are the datasets clearly presented in a useable and accessible format?

Not applicable

Reviewer Expertise:

Epidemiology, meningococcal disease, research synthesis

We confirm that we have read this submission and believe that we have an appropriate level of expertise to confirm that it is of an acceptable scientific standard.

## References

[1] : Impact of MenAfriVac in nine countries of the African meningitis belt, 2010–15: an analysis of surveillance data. *The Lancet Infectious Diseases* .2017;17(8) : 10.1016/S1473-3099(17)30301-8 867-872 10.1016/S1473-3099(17)30301-8 28545721

[2] : Description and nomenclature of Neisseria meningitidis capsule locus. *Emerg Infect Dis* .2013;19(4) : 10.3201/eid1904.111799 566-73 10.3201/eid1904.111799 23628376PMC3647402
